# Hepatitis C virus notification rates in Australia are highest in socioeconomically disadvantaged areas

**DOI:** 10.1371/journal.pone.0198336

**Published:** 2018-06-18

**Authors:** Samuel W. Hainsworth, Paul M. Dietze, David P. Wilson, Brett Sutton, Margaret E. Hellard, Nick Scott

**Affiliations:** 1 Disease Elimination Program, Burnet Institute, Melbourne, Victoria, Australia; 2 Behaviours and Health Risks, Burnet Institute, Melbourne, Victoria, Australia; 3 Department of Epidemiology and Preventive Medicine, Monash University, Clayton, Victoria, Australia; 4 Victorian Department of Health and Human Services, Melbourne, Victoria, Australia; 5 Department of Infectious Diseases, The Alfred Hospital and Monash University, Melbourne, Victoria, Australia; Duke University, UNITED STATES

## Abstract

**Background:**

Poor access to health services is a significant barrier to achieving the World Health Organization’s hepatitis C virus (HCV) elimination targets. We demonstrate how geospatial analysis can be performed with commonly available data to identify areas with the greatest unmet demand for HCV services.

**Methods:**

We performed an Australia-wide cross-sectional analysis of 2015 HCV notification rates using local government areas (LGAs) as our unit of analysis. A zero-inflated negative binomial regression was used to determine associations between notification rates and socioeconomic/demographic factors, health service and geographic remoteness area (RA) classification variables. Additionally, component scores were extracted from a principal component analysis (PCA) of the healthcare service variables to provide rankings of relative service coverage and unmet demand across Australia.

**Results:**

Among LGAs with non-zero notifications, higher rates were associated with areas that had increased socioeconomic disadvantage, more needle and syringe services (incidence rate ratio [IRR] 1.022; 95%CI 1.001, 1.044) and more alcohol and other drug services (IRR 1.019; 1.005, 1.034). The distribution of PCA component scores indicated that per-capita healthcare service coverage was lower in areas outside of major Australian cities. Areas outside of major cities also contained 94% of LGAs in the lowest two socioeconomic quintiles, as well as 35% of HCV notifications despite only representing 29% of the population.

**Conclusions:**

As countries aim for HCV elimination, routinely collected data can be used to identify geographical areas for priority service delivery. In Australia, the unmet demand for HCV treatment services is greatest in socioeconomically disadvantaged and non-metropolitan areas.

## Introduction

The availability of new tools and approaches for the treatment of hepatitis C virus (HCV), including highly effective direct-acting antiviral (DAA) treatments, has facilitated the development of the World Health Organization’s (WHO) HCV elimination targets. These targets propose an 80% reduction in HCV incidence and a 65% reduction in HCV-related mortality by 2030 [1]. Australia is currently one of the few countries where all people living with HCV can access DAA treatments, due to the Australian government committing over AU$1 billion over five years for an unlimited number of treatments, with no restriction on access according to disease stage, treatment history or drug use status [2–4]. This landmark decision is a major step towards achieving HCV elimination.

Similar to other industrial countries, people who inject drugs (PWID) are the group at greatest risk of transmission and infection in Australia [5]. Modelling suggests that treating 4,700 PWID per year for 15 years would enable Australia to reach the WHO elimination targets [6]; however, testing will also need to be significantly scaled up in patients’ communities to achieve this treatment target [7]. Hence, it will be vitally important that health services throughout the country–city, regional and rural–have the capacity to find, link and engage PWID in care to generate sufficient treatment demand.

There is concern that current services to prevent, diagnose and treat HCV are not distributed equitably across Australia and that services and infrastructure will need to be improved in some areas. Ideally this should include access to a mix of harm reduction services for PWID (e.g. clean needles and syringes and drug counselling services) and to general practitioners (GPs) who have received up to date information on HCV testing and the availability of DAA treatments for HCV–in particular the capacity of GPs to prescribe them to patients without requiring specialist referral [8], provided there are no additional complications (e.g. liver cirrhosis or coinfection with hepatitis B or HIV). For patients with additional complications, access to specialist services and/or hospitals without requiring a burdensome amount of travel is critical for the appropriate management of HCV. Without an equitable geographic distribution of these services it is possible that some areas will be left behind as Australia attempts to reach the WHO elimination targets for HCV.

Despite this, we are unaware of any studies of the geographical distribution of people living with HCV relative to HCV-related health services, information which is critical to inform needs-based planning and effective resource allocation. This study aimed to identify and quantify the relationships between the geographical distribution of HCV notifications and socioeconomic/demographic factors, health service coverage and geographic remoteness. These are important issues to consider in needs-based service planning [9]. Identifying the key characteristics of high-priority regions will inform the equitable distribution of HCV resources and the scale-up of testing and treatment programs. More broadly, this paper provides a general framework that can be readily replicated by researchers in other countries and areas of public health in order to leverage insights from commonly available data.

## Methods

### Data collation and description

#### Outcome variable

HCV is a notifiable disease in Australia. This means that all positive tests are reported to the Australian Government Department of Health (DoH) as notification. For the purposes of this paper we define a HCV notification as a positive test reported to the DoH. We obtained anonymised data of the number of HCV notifications per month in 2015 from the DoH, matched to postcode by place of residence [10].

#### Unit of geographic analysis

Australian postcodes can cover several non-adjacent suburbs, meaning that contiguous geographic regions cannot be identified by a postcode alone. Therefore, we used local government areas (LGAs), which are 545 contiguous and non-overlapping administrative units covering all of Australia, as our unit of analysis.

A postcode-to-LGA mapping scheme was devised using Australian Bureau of Statistics (ABS) correspondence data in which single postcodes can map to multiple LGAs weighted by 2016 population data [11]. Assuming notifications were uniformly distributed across a postcode’s population, the percentage of a postcode’s HCV notifications distributed between LGAs was determined by the percentage of its population in that LGA. For example, if an LGA contained 95% of a postcode’s population, it was allocated 95% of its notifications. All concordances were calculated in relation to 2016 LGA classifications in order to minimise the impact of boundary changes that occur to LGAs over time.

#### Predictor variables

Profiles for each LGA were built through collation of data from a variety of sources. ABS data were used to estimate the population size [12], the proportion of the population of Aboriginal or Torres Strait Islander (ATSI) descent [13], the proportion of the population born overseas [12], Index of Relative Socioeconomic Disadvantage (IRSD) quintiles [14] and the number of prisons [15–19]. We included the proportion ATSI in the population because this group is known to have disproportionately high HCV prevalence compared to the general population [20, 21]. The distribution of this variable is shown in S1 Fig. IRSD quintiles were ranked in order of increasing socioeconomic advantage, with quintile 1 indicating the 20% most disadvantaged LGAs in Australia. Remoteness area classification (RA) were also obtained from the ABS [22]. The RA category of ‘major city’ represents relatively unrestricted access to a wide range of goods, services and opportunities for social interaction, with subsequent categories indicating progressively diminished access.

We also obtained a number of general and HCV-specific healthcare variables. The total number of needle and syringe programs (NSPs) as well as NSPs open during business hours (including primary and secondary sites and vending machines) [23–29]. Data from the Australian Urban Research Infrastructure Network [30] were used to calculate the number of GP clinics, alcohol and other drug (AOD) services, hospitals and liver specialists in each LGA. Table 1 provides a description of the primary services offered by each healthcare service included in our analysis.

**Table 1:** Primary services for HCV.

Service	Primary services
**NSP**	Prevention
**AOD**	Prevention/testing
**GP**	Testing/treatment
**Specialist**	Treatment
**Hospital**	Treatment

#### Time frame

Data were collected to best represent LGAs during 2015. The number of GPs, AOD services, hospitals and liver specialists were all provided for 2015; however, RA, IRSD, proportion of population ATSI, and proportion of population born overseas were reported for 2011 only, and the number of NSPs were collected for 2016. These variables were used to approximate LGA characteristics in 2015. It is possible, though unlikely, that the March 2016 availability of DAA treatments for HCV may have affected the 2016 NSP variables.

#### Notification rates

To estimate notification rates and to control for population size, notification counts were normalised by LGA population size and scaled by a constant (10^5^). This quantity represents the notification rate per 100,000 population in an LGA. Because the number of individuals exposed to testing was unknown, this estimate was used as a proxy for the true notification rate (i.e. the number of notifications per person subject to testing). The data were rounded to the nearest integer for appropriate use as count data.

In areas with small population sizes, notification rates are sensitive to small changes in the number of notifications and are therefore susceptible to greater rate variance than areas with larger populations. We observed 55 LGAs with artificially inflated rates which we removed from our analysis (S2 Fig).

**Fig 1:**
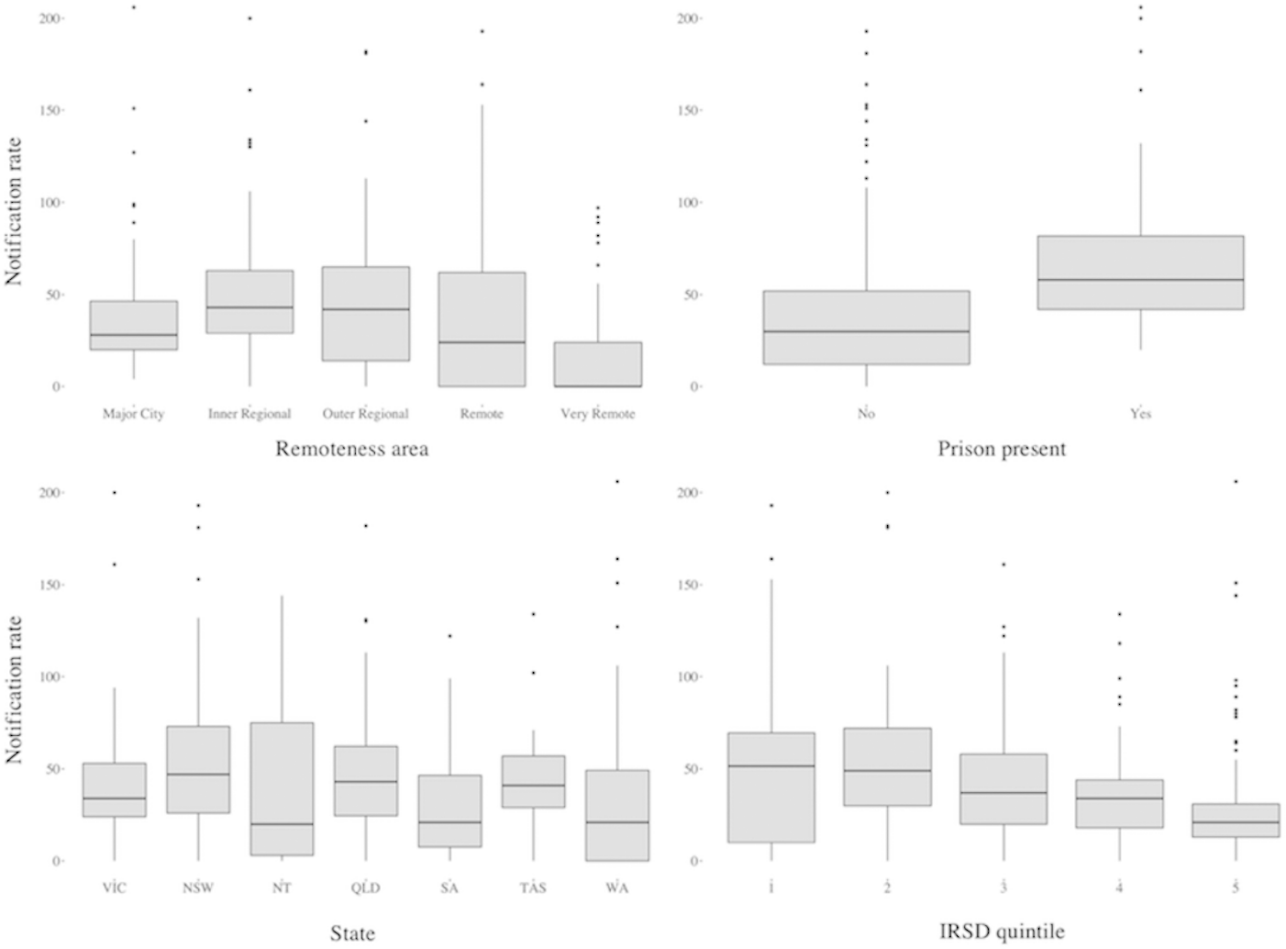
**Bivariate distribution of notification rate (per 100,000 population) and remoteness areas (top left), presence of a prison (top right), state (bottom left) and IRSD quintile (bottom right).** The vertical axis has been cropped to 200 notifications per 100,000 population in each plot to improve readability.

Fig 1 shows the distribution of notification rates according to some key predictor variables and is suggestive of underlying correlations.

### Statistical methods

**Fig 2:**
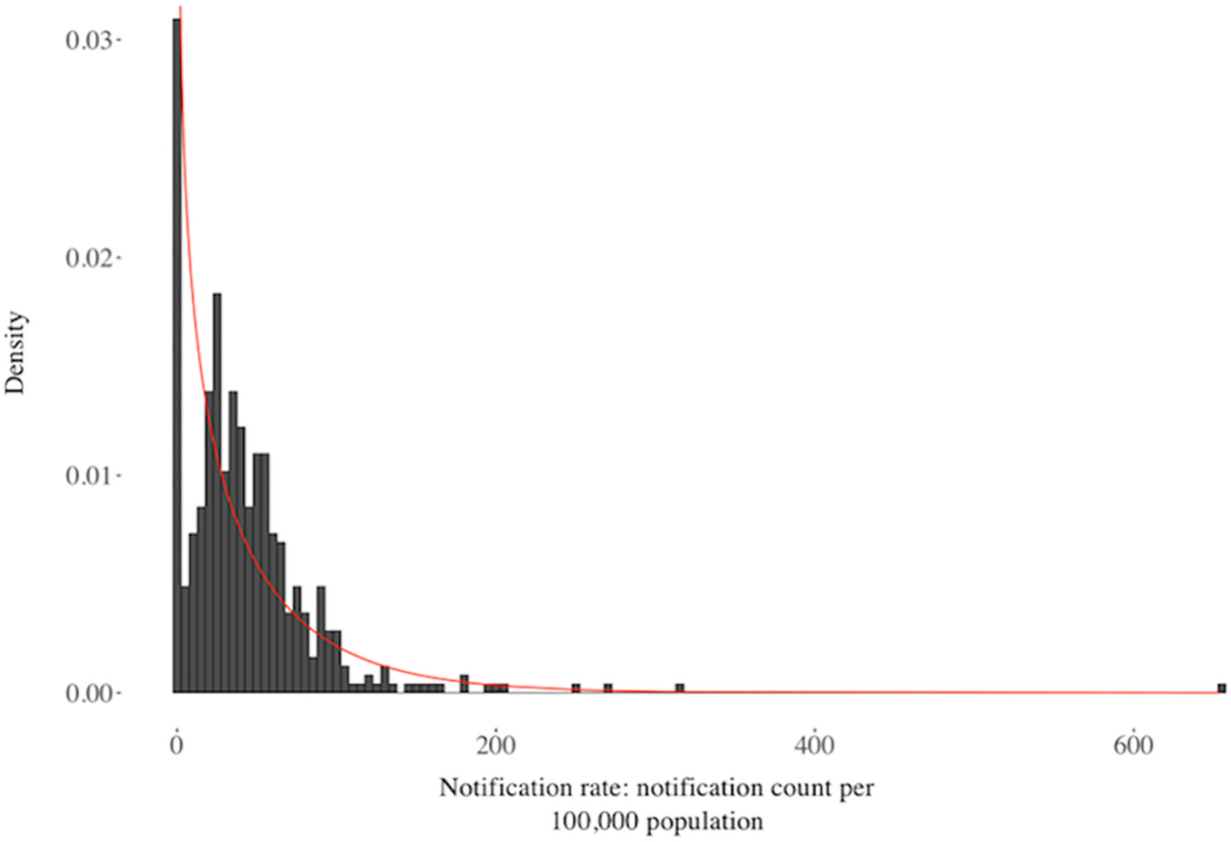
Univariate distribution of annual notifications per 100,000 population. A negative binomial curve is overlaid (red), with a mean of 43.1 (the empirical mean) and dispersion of 0.70.

Zero-inflated models are useful tools for describing data which may come from two separate data generating processes, treating the observation of zero and non-zero data independently. A HCV notification requires two conditions; first the presence of HCV in the area and, second, access to testing services. Observing a non-zero rate is, therefore, described by a different generation process than a zero rate. Moreover, our data contained an excess of zero rates (Fig 2) and was over-dispersed (the variance was larger than the mean by a factor of six). Consequently, we fitted a zero-inflated negative binomial model to our chosen predictors, with 2015 notification rates from 490 remaining LGAs as the outcome variable. Our model had two components: one that determined the relationships between LGAs and zero notification rates, and a second that fitted a negative binomial model to the non-zero rates. Vuong’s non-nested hypothesis test confirmed that this model provided a better fit to the data than a standard negative binomial model [31].

Subsequently, we derived a measure for the availability of services to prevent, diagnose and treat HCV. Following a similar procedure to Dietze et al. [9], we performed a principal components analysis (PCA) on the unscaled covariance matrix of a subset of predictors related to universal and HCV-specific healthcare services (number of NSPs, GPs, hospitals, specialists and AOD services) and selected the component onto which the healthcare service variables loaded most strongly. The extracted principal component and associated component scores of each LGA were scaled by population size and interpreted as the relative healthcare service coverage per capita. Notification rates and component scores were then combined to provide a metric for the unmet demand for services.

#### Services in neighbouring LGAs

To investigate the potential impact of services in nearby LGAs, the number of health services and NSPs in neighbouring LGAs within a 50km radius of each LGA’s geographic centre were initially included in the regression. Since this did not improve the fit of the model, these variables were omitted from the study.

#### LGAs without prisons

Because PWID are over-represented among prison populations [32], LGAs containing prisons were expected to have higher than average notification rates. However, since prisoners do not use the same health services as the wider population, there was a possibility that these LGAs could skew the results. Therefore, the model was also fit to LGAs that did not contain a prison, but this made no significant difference and these LGAs were included in the final analysis.

## Results

### Distribution of notification rate data

**Fig 3:**
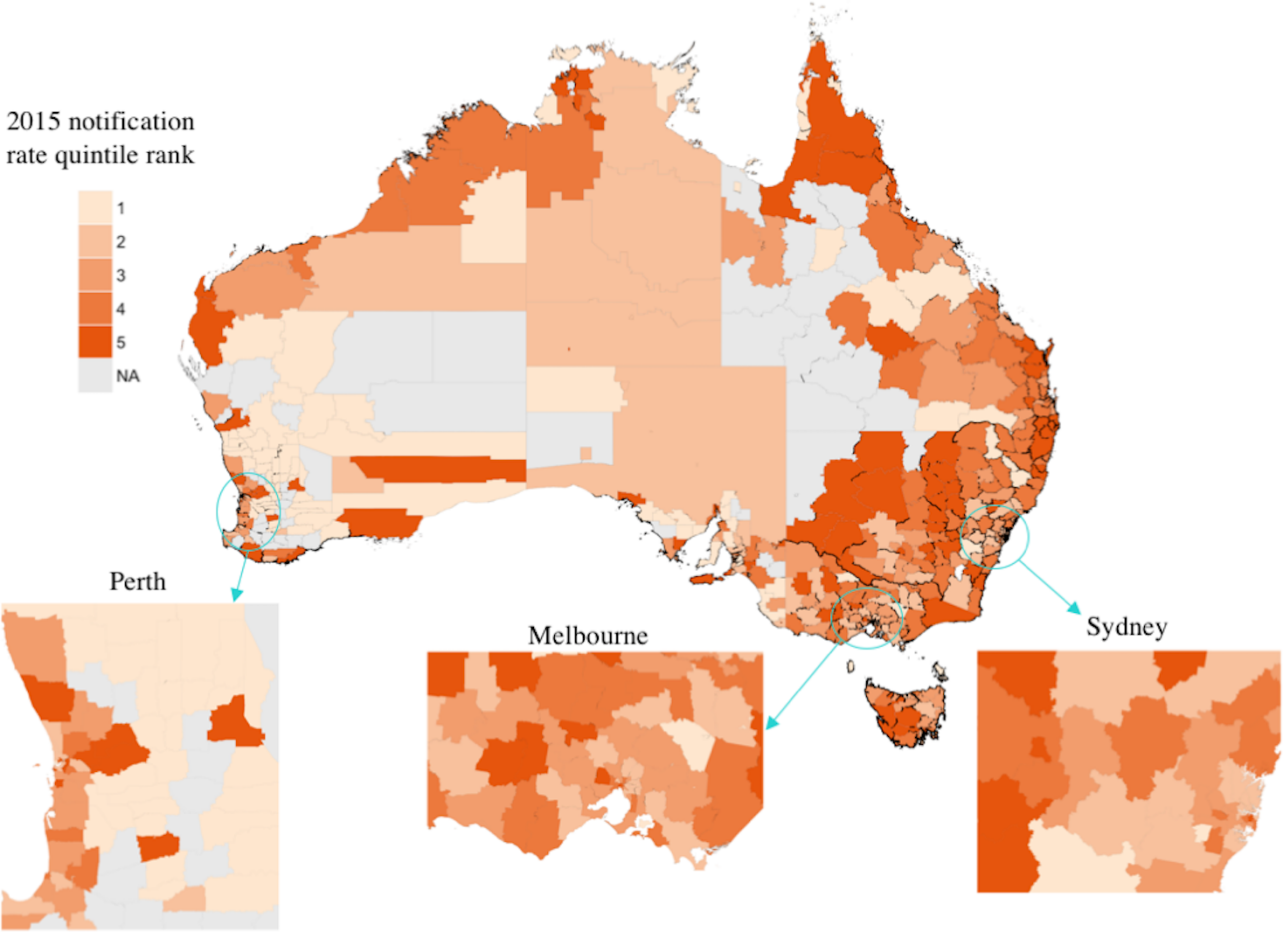
Quintile rank of 2015 notification rates by Australian Local Government Area. Quintile 1 indicates the 20% of LGAs with the lowest notifications per 100,000 population. LGAs removed from the analysis are indicated in grey. Adapted from [33] under a CC BY license, with permission from the Commonwealth of Australia, original copyright 2016.

During 2015 35% of all HCV notifications were outside major cities, while 32% were from either inner or outer regional areas, despite only 29% of the population residing outside major cities and 25% in regional areas. Fig 3 visualises the geospatial distribution of notification rate quintiles (the geospatial distribution of notification counts is provided in the supplementary material–S3 Fig). LGAs with the highest rates tended to be outside of metropolitan areas, particularly in New South Wales, Queensland and Victoria. Fig 2 illustrates the high degree of positive skew of the notification rate variable, characteristic of count data. The negative binomial distribution consistently underestimates rates between 0 and 100, while overestimating rates between 100 and 200.

### Zero-inflated negative binomial model

The results of the model are shown in Table 2. The negative binomial component of the model fitted the LGAs with non-zero notifications per 100,000 population. Higher notification rates were associated with LGAs that had increased socioeconomic disadvantage (lower IRSD quintiles), more NSP services (incidence rate ratio [IRR] 1.022; 95% CI 1.001, 1.044), more AOD services (IRR 1.019; 1.005, 1.034), the presence of a prison (1.606; 1.359, 1.896) and were in the states of New South Wales (IRR 1.475; 1.201, 1.811), Queensland (IRR 1.487; 1.137, 1.943) and Western Australia (IRR 1.554; 1.209, 1.997) compared to Victoria. While the model detected notification rates to be statistically significantly lower in LGAs with more GPs, the size of the association was small (IRR 0.998; 0.996, 1.000).

**Table 2:** Zero-inflated negative binomial regression.

	Count Model			Zero-Inflated Model		
Coefficient	Estimate (Incidence rate ratio)	95% CI	p-value	Estimate (Odds ratio)	95% CIs	p-value
(Intercept)	33.266	(22.382, 49.444)	<0.001*	––^b^	––	––
Number of NSPs	1.022	(1.001, 1.044)	0.042*	0.904	(0.600, 1.363)	0.631
IRSD Quintile 2	1.026	(0.833, 1.263)	0.812	1.940	(0.357, 10.561)	0.443
IRSD Quintile 3	0.725	(0.578, 0.909)	0.005*	1.860	(0.328, 10.51)	0.485
IRSD Quintile 4	0.601	(0.475, 0.761)	<0.001*	3.520	(0.563, 22.044)	0.178
IRSD Quintile 5	0.456	(0.352, 0.592)	<0.001*	2.040	(0.309, 13.503)	0.459
Outside major city	1.196	(0.957, 1.493)	0.115	––^b^	––	––
Proportion born overseas	1.007	(0.999, 1.015)	0.101	0.960	(0.903, 1.02)	0.187
Number of GPs	0.998	(0.996, 1.000^a^)	0.024*	0.688	(0.565, 0.837)	<0.001*
Specialists	1.000	(0.998, 1.001)	0.642	0.878	(0.770, 1.001)	0.052
Hospitals	1.005	(0.991, 1.02)	0.468	1.430	(1.064, 1.912)	0.018*
Drug Services	1.019	(1.005, 1.034)	0.010*	0.930	(0.713, 1.212)	0.589
Proportion ATSI	0.998	(0.992, 1.004)	0.448	1.020	(0.989, 1.055)	0.202
NSW	1.475	(1.201, 1.811)	<0.001*	0.092	(0.007, 1.157)	0.065
NT	1.402	(0.912, 2.156)	0.123	8.690	(0.405, 186.39)	0.167
QLD	1.487	(1.137, 1.943)	0.004*	2.220	(0.265, 18.579)	0.462
SA	0.826	(0.653, 1.046)	0.112	1.970	(0.341, 11.355)	0.449
TAS	1.164	(0.868, 1.559)	0.310	0.369	(0.039, 3.488)	0.384
WA	1.554	(1.209, 1.997)	0.001*	4.840	(0.786, 29.834)	0.089
Prison present	1.606	(1.359, 1.896)	<0.001*	––^b^	––	––

The output is decomposed into estimates for the negative binomial count model and the zero-inflated model. Coefficient estimates have been exponentiated, along with 95% CIs and the associated p-value. Statistically significant estimates are denoted by (*).

^a^ Coefficient is statistically significant; however rounded up to 1.000 to three decimal places.

^b^ An accurate baseline likelihood of a zero rate could not be determined because there was insufficient data in our binary variables: of the LGAs with zero counts, all were outside major cities and none had a prison.

Geographical remoteness was not significantly associated with notification rates in the model; however, the data showed that 94% of LGAs in the two most socioeconomically disadvantage quintiles are outside of major cities, suggesting that socioeconomic disadvantage is highly correlated with geographical remoteness and therefore may remain undetected by the regression model.

The zero-inflated component of the model fitted the LGAs with zero rates. The coefficient estimates indicated that zero rates were more likely for LGAs with more hospitals (odds ratio [OR] 1.430; 95% CI 1.064, 1.912) and less likely for LGAs with more GPs (OR 0.688; 0.565, 0.837 for every additional GP).

### Healthcare service coverage component scores

**Fig 4:**
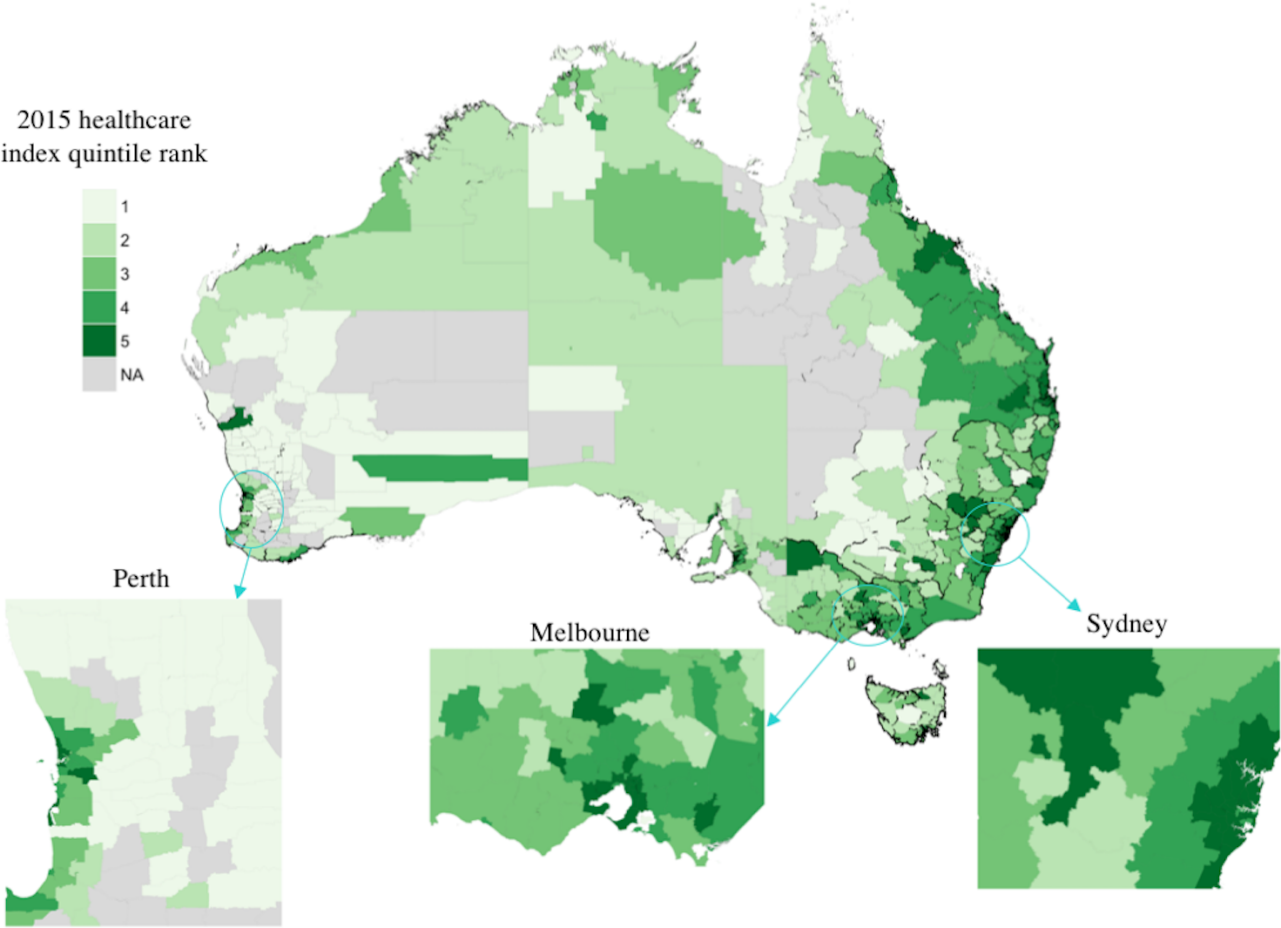
Quintile rank of 2015 relative healthcare coverage index by Australian Local Government Area. Quintile 1 indicates the 20% of LGAs with lowest healthcare service scores. LGAs removed from the analysis are indicated in grey. Adapted from [33] under a CC BY license, with permission from the Commonwealth of Australia, original copyright 2016.

The selected principal component captured 87% of the variation in the healthcare data (Table 3). Larger variable loadings for treatment services (specialists, hospitals, GPs) indicated greater variance in the number of these services than in prevention or testing services (NSPs, AOD). High coverage scores were, therefore, more likely to indicate high numbers of treatment services than prevention or testing services. Fig 4 shows the distribution of quintile ranks for these component scores across Australian LGAs. Of the non-metropolitan LGAs, 54% were ranked in the lowest two healthcare quintiles nationally, while 90% of metropolitan areas were in the highest two quintiles nationally. This suggests that non-metropolitan areas may have the lowest treatment service coverage per capita.

**Table 3:** Loadings for healthcare services variables onto the first principle component extracted from the PCA.

Variable	Loading
**NSPs**	0.347
NSPs after hours	0.190
Hospitals	0.846
GPs	0.876
Drug and alcohol services	0.728
Specialists	0.970

### Unmet demand

**Fig 5:**
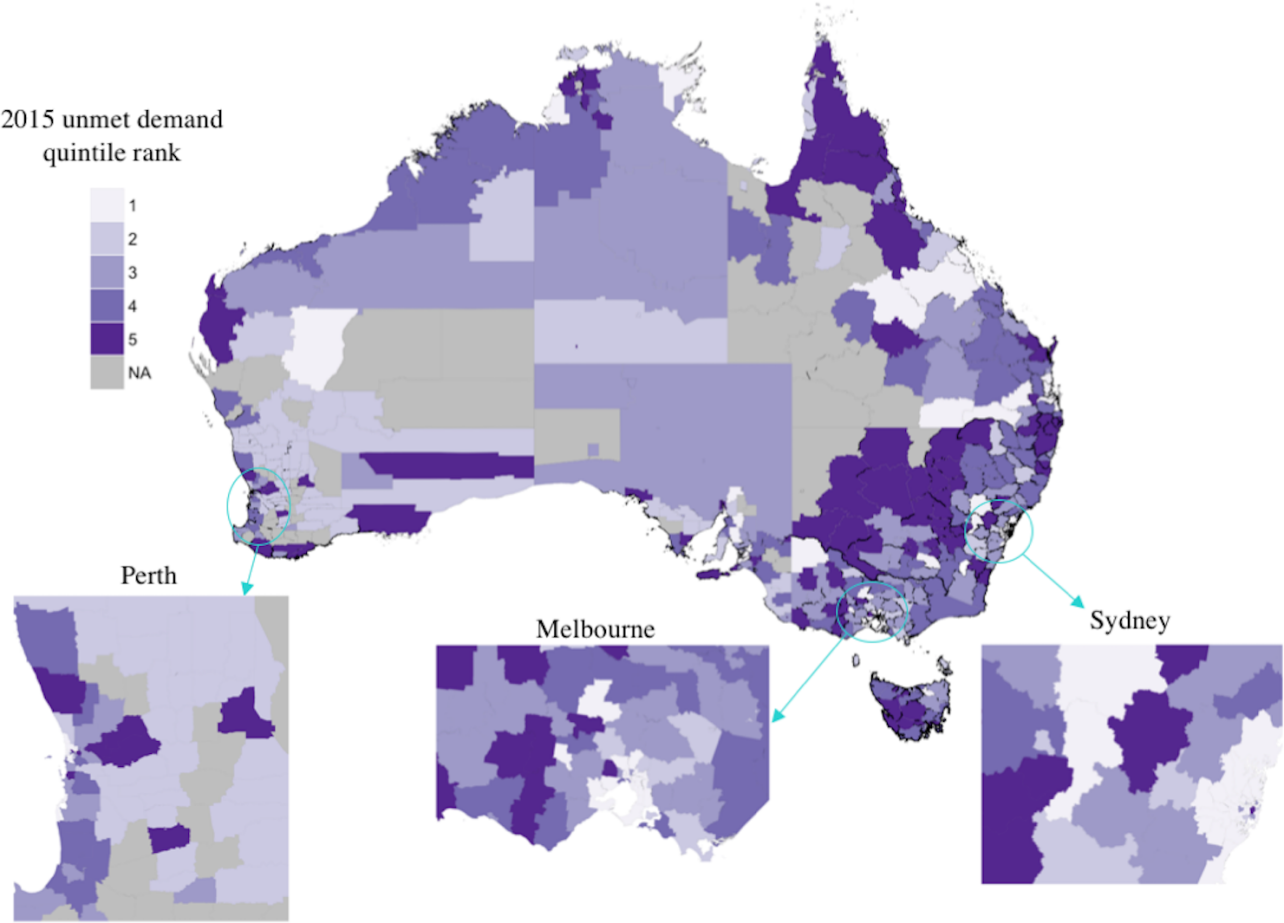
Quintile rank of 2015 unmet demand index by Australian Local Government Area. Quintile 1 indicates the 20% of LGAs with the least unmet demand. LGAs removed from the analysis are indicated in grey. Adapted from [33] under a CC BY license, with permission from the Commonwealth of Australia, original copyright 2016.

The unmet demand index of an LGA was defined to be the difference between standardised notification rates and standardised healthcare coverage scores. Fig 5 shows the distribution of quintile ranks for the unmet demand index; comparison with Figs 3 and 4 shows the greatest unmet demand occurs primarily where there is simultaneously a high per-capita HCV burden and low relative healthcare coverage. These areas are predominantly non-metropolitan.

## Discussion

This study found evidence suggesting that HCV burden is inequitably distributed across Australia. Among LGAs with at least one notification, notification rates were, on average, higher in areas that had greater socioeconomic disadvantage, more NSP services, more AOD services, at least one prison and were in the states of New South Wales, Queensland and Western Australia, relative to Victoria. When notification rates were assessed against a measure of per-capita healthcare service coverage, we found the unmet demand for services to be greatest in geographic areas outside major cities, which included a disproportionate amount of socioeconomically disadvantaged LGAs compared to major cities.

While we cannot be certain about the drivers behind this behaviour, we hypothesise that unequal distribution of notifications may be explained by inadequate healthcare coverage for the prevention and treatment of HCV. We observed inequity in the geographic distribution of notified HCV cases, as 35% were from patients residing outside major cities of Australia. It is unlikely that remote areas have the greatest burden, since Gibney et. al. [34] has found notification incidence is higher in non-remote areas than remote areas, and 32% of all notifications were from inner or outer regional areas as opposed to only 3% in remote areas. It may be, therefore, that the greatest HCV burden occurs in inner and outer regional, as opposed to remote or metropolitan, areas.

Previous studies have identified non-metropolitan areas of Australia as having greater socioeconomic disadvantage and lower healthcare access for multiple health-related issues [35, 36], including insufficient access to healthcare facilities for vulnerable people living in these areas [37]. It has also been shown that socioeconomic disadvantage is a significant risk factor for HCV [34], consistent with our regression results. The index for unmet demand, developed in this study, indicated that areas experiencing high notification rates of HCV tend to be non-metropolitan areas with poor healthcare coverage. Specifically, coverage of primary treatment services is low, possibly suggesting that patients are being tested and notified in these areas but have inadequate engagement with treatment programs to prevent further spread of HCV infection. As such, there is a need to ensure we have adequate HCV support services in place to find and link patients into care in order to achieve 2030 elimination targets.

Additional barriers to engagement with care and HCV treatment completion exist, such as the stigma associated with both illicit drug use and HCV infection [38, 39]. Previous research has identified the attitudes and behaviours of staff as a key factor in improving patient engagement [40]. If the healthcare workforce in rural and regional Australia is under-resourced, with limited time to work with patients with complex health needs [41], this has the potential to exacerbate the problem of under-engagement of PWID. Hence, the scale-up of services in these areas should be accompanied by resourcing to educate and support healthcare staff work with PWID and people with complex health and social issues.

Higher notification rates were observed in areas with a greater number of NSP and AOD services. These services are generally targeted to areas with increased drug market activity, which is a known driver of HCV transmission and may offer an explanation for this association. Moreover, NSP and AOD services often provide referral information for clients to HCV testing and treatment services, in particular those that may be viewed as more culturally acceptable for PWID. It is therefore possible that NSP and AOD services may have facilitated increased testing among PWID, leading to higher notification rates.

Despite ATSI populations having a disproportionately high HCV prevalence compared to the general population [20, 21], we did not observe a correlation between notification rates and LGAs with large ATSI populations. Possible explanations for this include poor access to services and hence low levels of testing in these areas, or as has been reported anecdotally, the high levels of stigma and shame associated with injecting drugs and HCV in this population could be preventing people from seeking testing and treatment [42].

### Limitations

These findings are based on HCV notifications, as opposed to incidence. The absence of a relationship between notification rates and areas with greater ATSI populations may be a direct consequence of this since we cannot detect undiagnosed infections. It may be that these areas require targeted testing campaigns. In addition, because we did not have access to testing or treatment data, we could only speculate on the true drivers of notification rate behaviour.

The zero-inflated component of the model suffered from large uncertainty coefficient estimates caused by the absence of one of the two categories in the binary RA and prisons variables. This uncertainty made inference about the relationships between covariates and zero rates less reliable, as reflected in the 95% confidence intervals.

The modelling approach has two key limitations. First, the model does not account for all factors that may influence HCV notifications, as some factors were unavailable or could not be directly measured. Second, using LGAs as our unit of analysis (which cover very large geographic areas) resulted in poor resolution for our analysis and, consequently, we could only model high-level factors related to HCV. At this scale, even adjacent LGAs could vary substantially in demographics and healthcare service access. Fig 3 illustrates this poor resolution, showing a high degree of rate variability between some adjacent LGAs. Nevertheless, this model provides a qualitative description of some of the issues related to HCV notifications per capita.

## Conclusions

High-coverage treatment scale-up is required if Australia is to eliminate HCV as a public health threat, and this scale-up is necessary now if we are to achieve the WHO elimination targets by 2030. While this is only a preliminary analysis of the HCV burden, the findings provide important information for service prioritisation and planning, highlighting that the per capita burden of HCV is greatest in socioeconomically disadvantaged areas and the unmet demand for HCV services is greatest in geographic areas outside major cities. Our results suggest that strategies for HCV prevention and treatment in Australia would benefit from considering these factors. Any future research which has access to testing and treatment data could provide greater insight for the development of needs-based service planning. Despite this, the data used in our analysis are routinely available demographic and health service data, meaning that our analysis can serve as a useful framework for the ongoing monitoring of Australia’s efforts to eliminate HCV. Additionally, a similar framework for service planning could be employed in other countries wanting to scale up HCV testing and treatment in an effort to achieve HCV elimination.

## Supporting information

S1 File Discussion of supporting information figures. (DOCX)

S2 File Data availability information. (DOCX)

S1 Fig 2011 proportion ATSI by Australian Local Government Area. Adapted from [33] under a CC BY license, with permission from the Commonwealth of Australia, original copyright 2016.
(TIF)

S2 Fig Notification rate plotted against population size quintile (left) and notification count quintile (right). Quintile 1 indicates the 20% of LGAs with the lowest populations or counts. The vertical axis has been cropped to 200 notifications per 100,000 population in each plot to improve readability.
(TIF)

S3 Fig 2015 HCV notification counts by Australian Local Government Area. Adapted from [33] under a CC BY license, with permission from the Commonwealth of Australia, original copyright 2016.
(TIF)

S4 Fig 2015 population quintiles by Australian Local Government Area. Quintile 1 indicates the 20% of LGAs with the lowest population. Adapted from [33] under a CC BY license, with permission from the Commonwealth of Australia, original copyright 2016.
(TIF)

## Abbreviations

AOD: alcohol and other drug
ATSI: Aboriginal and Torres Strait Islander
DAA: direct-acting antiviral
GP: general practitioner
HCV: hepatitis C virus
IRSD: Index of Relative Socioeconomic Disadvantage
LGA: local government area
NSP: needle and syringe program
NSW: New South Wales
OST: opioid substitution therapy
PCA: principle components analysis
PWID: people who inject drugs
QLD: Queensland
RA: remoteness area
VIC: Victoria
WA: Western Australia
WHO: World Health Organization
