# Supplementary material for: Hepatitis C virus notification rates in Australia are highest in socioeconomically disadvantaged areas
Source: PLoS One. 2018 Jun 18;13(6):e0198336. doi: 10.1371/journal.pone.0198336 (PMC6005510; doi:10.1371/journal.pone.0198336)
Supplement: S1 File — (DOCX) [file pone.0198336.s001.docx]

**Supporting information**

**ATSI distribution**

**S1 Fig. 2011 proportion ATSI by Australian Local Government Area.** Adapted from [33] under a CC BY license, with permission from the Commonwealth of Australia, original copyright 2016.

**Variance of notification rates**

If a significant number of LGAs with small population sizes had higher notification rates than areas with larger populations, this could bias our results. To better understand if this was the case, we produced two plots (S2 Fig, while S4 Fig shows Australia’s population distribution). The left plot illustrates that rate variation decreases with increasing population size, as expected. LGAs with the lowest populations showed significant skew toward a rate of zero, but with large variance, having a median of 0 and a standard deviation of 68 notifications per 100,000 population. To account for this, LGAs which belonged to this quintile and had a nonzero notification count were removed from our analysis.

**S2 Fig. Notification rate plotted against population size quintile (left) and notification count quintile (right).** Quintile 1 indicates the 20% of LGAs with the lowest populations or counts. The vertical axis has been cropped to 200 notifications per 100,000 population in each plot to improve readability.

**Notification counts**

S3 Fig shows that the highest notification counts are clustered around the cities of Melbourne, Sydney and Brisbane.

**S3 Fig. 2015 HCV notification counts by Australian Local Government Area.** Adapted from [33] under a CC BY license, with permission from the Commonwealth of Australia, original copyright 2016.

**Population distribution**

S4 Fig shows that the most densely populated LGAs are located on the South coast of Victoria and along the East coast of NSW and Queensland, corresponding to the location of the cities of Melbourne, Sydney and Brisbane, respectively.

**S4 Fig. 2015 population quintiles by Australian Local Government Area.** Quintile 1 indicates the 20% of LGAs with the lowest population. Adapted from [33] under a CC BY license, with permission from the Commonwealth of Australia, original copyright 2016.
