# Supplementary material for: Hepatitis C virus notification rates in Australia are highest in socioeconomically disadvantaged areas
Source: PLoS One. 2018 Jun 18;13(6):e0198336. doi: 10.1371/journal.pone.0198336 (PMC6005510; doi:10.1371/journal.pone.0198336)
Supplement: S2 File — (DOCX) [file pone.0198336.s002.docx]

**Data availability statement**

All the data used in this study can be found through the links provided below or by requesting access through the provided contact information.

**Australia-wide hepatitis C notifications**

To obtain this data, a request should be sent to the Australian Department of Health and Human Services (DHHS). Requests may be sent by email to [enquiries@dhhs.vic.gov.au](mailto:enquiries@dhhs.vic.gov.au) or through their website (https://dhhs.vic.gov.au/contact). Alternatively, a request can be sent to Nick Scott at the Burnet Institute (email: [nick.scott@burnet.edu.au](mailto:nick.scott@burnet.edu.au)).

**Number of general practitioners, alcohol and other drug services, hospitals and liver specialists across Australia.**

Access to this data can be requested at the following link

<https://aurin.org.au/national-health-services-directory-restricted-data-set-application/>

**Population size by Australian local government area**

<http://www.abs.gov.au/AUSSTATS/abs@.nsf/DetailsPage/3218.02014-15?OpenDocument>

**Number of Aboriginal or Torres Strait Islander Australians**

<http://www.abs.gov.au/AUSSTATS/abs@.nsf/DetailsPage/3238.0.55.001June%202011?OpenDocument>

**Number of Australians born overseas**

<http://www.abs.gov.au/AUSSTATS/abs@.nsf/DetailsPage/3218.02014-15?OpenDocument>

**The Index of Relative Socio-Economic Disadvantage**

<http://www.abs.gov.au/websitedbs/censushome.nsf/home/seifa>

**The number of prisons (various sources by state or territory)**

New South Wales

<http://www.correctiveservices.justice.nsw.gov.au/Pages/CorrectiveServices/custodial-corrections/table-of-correctional-centres/correctional-centres.aspx>

Northern Territory

<https://nt.gov.au/law/prisons>

Queensland

<https://www.qld.gov.au/law/sentencing-prisons-and-probation/prisons-and-detention-centres/prison-locations/>

South Australia

<http://www.corrections.sa.gov.au/sa-prisons>

Tasmania

<http://www.justice.tas.gov.au/prisonservice/visiting>

Victoria

<http://www.corrections.vic.gov.au/home/prison/>

Western Australia

http://www.correctiveservices.wa.gov.au/prisons/prison-locations/default.aspx

**Number of needle and syringe programs**

New South Wales

<http://www.health.nsw.gov.au/hepatitis/Pages/nsp-outlets.aspx>

Northern Territory

<https://www.ntahc.org.au/programs/harm-reduction-needle-and-syringe-program/collection-and-disposal#accordion-0-0>

Queensland

<https://www.health.qld.gov.au/qnsp>

South Australia

http://www.sahealth.sa.gov.au/wps/wcm/connect/public+content/sa+health+internet/health+services/drug+and+alcohol+services/clean+needle+program

Tasmania

<http://www.dhhs.tas.gov.au/publichealth/communicable_diseases_prevention_unit/infectious_diseases/needle_and_syringe_program>

Victoria

<https://www2.health.vic.gov.au/alcohol-and-drugs/aod-treatment-services/aod-prevention-harm-reduction/needle-and-syringe-program>

Western Australia

<http://healthywa.wa.gov.au/Articles/U_Z/Where-to-find-needle-and-syringe-programs-in-WA>

**Remoteness areas**

<http://www.abs.gov.au/websitedbs/D3310114.nsf/home/remoteness+structure>

**Geographical correspondences**

<http://www.abs.gov.au/websitedbs/d3310114.nsf/home/correspondences>
