# Supplementary figures and images for: Hepatitis C virus notification rates in Australia are highest in socioeconomically disadvantaged areas
Source: PLoS One. 2018 Jun 18;13(6):e0198336. doi: 10.1371/journal.pone.0198336 (PMC6005510; doi:10.1371/journal.pone.0198336)

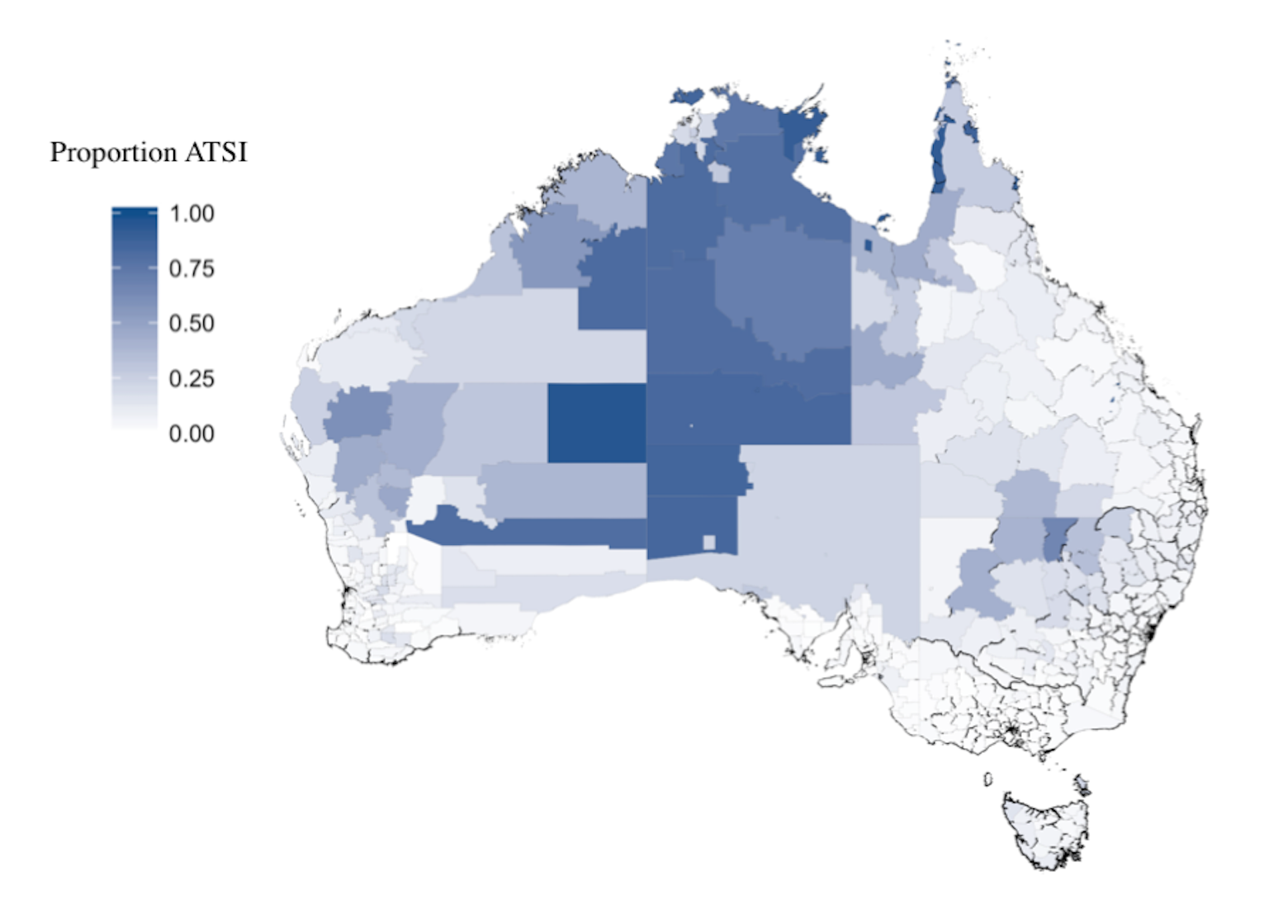

Supplement: S1 Fig — Adapted from [33] under a CC BY license, with permission from the Commonwealth of Australia, original copyright 2016. (TIF) [file pone.0198336.s003.tif]

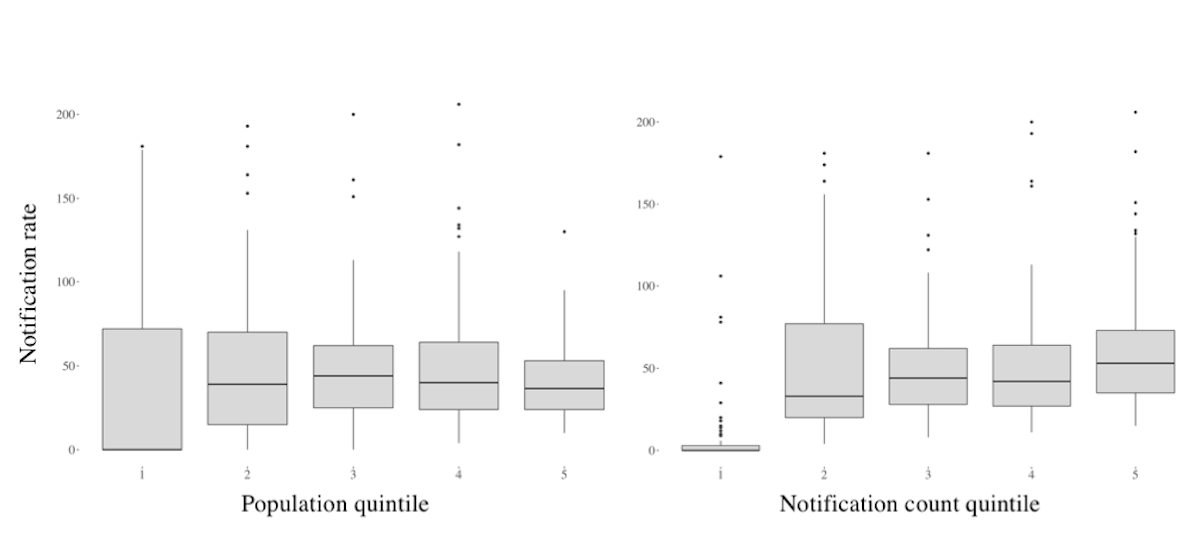

Supplement: S2 Fig — Quintile 1 indicates the 20% of LGAs with the lowest populations or counts. The vertical axis has been cropped to 200 notifications per 100,000 population in each plot to improve readability. (TIF) [file pone.0198336.s004.tif]

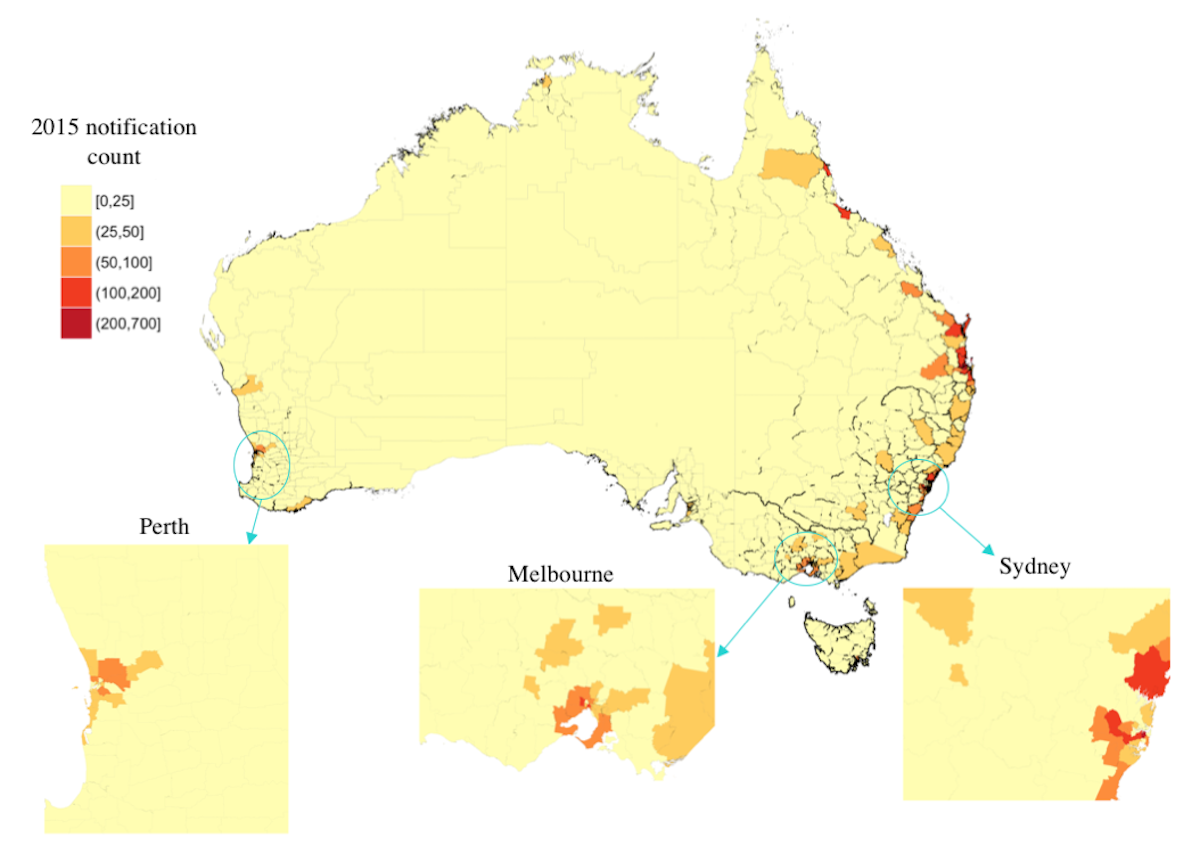

Supplement: S3 Fig — Adapted from [33] under a CC BY license, with permission from the Commonwealth of Australia, original copyright 2016. (TIF) [file pone.0198336.s005.tif]

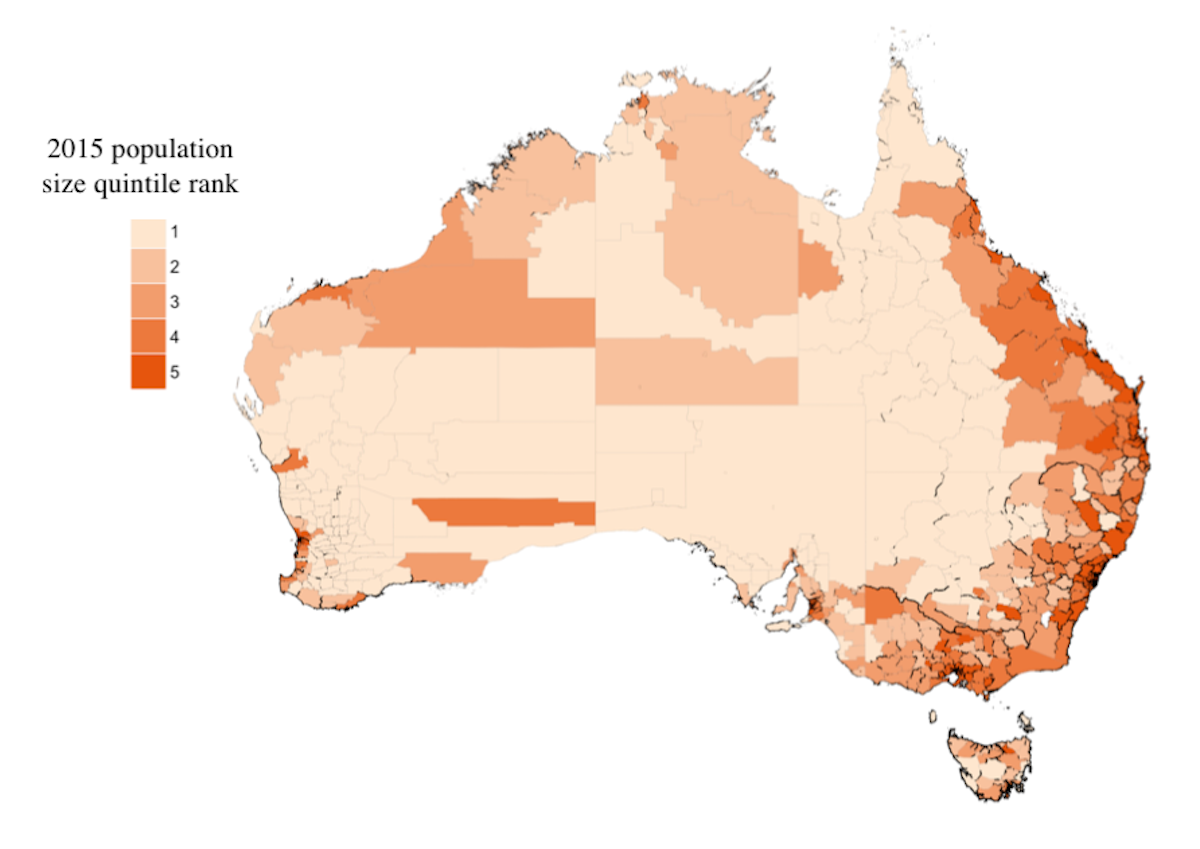

Supplement: S4 Fig — Quintile 1 indicates the 20% of LGAs with the lowest population. Adapted from [33] under a CC BY license, with permission from the Commonwealth of Australia, original copyright 2016. (TIF) [file pone.0198336.s006.tif]
